# The Cell Differentiation of Idioblast Myrosin Cells: Similarities With Vascular and Guard Cells

**DOI:** 10.3389/fpls.2021.829541

**Published:** 2022-01-10

**Authors:** Makoto Shirakawa, Mai Tanida, Toshiro Ito

**Affiliations:** Division of Biological Science, Graduate School of Science and Technology, Nara Institute of Science and Technology, Ikoma, Japan

**Keywords:** cell differentiation, FAMA, glucosinolate, guard cell, myrosin cell, myrosinase, vascular cell

## Abstract

Idioblasts are defined by abnormal shapes, sizes, and contents that are different from neighboring cells. Myrosin cells are Brassicales-specific idioblasts and accumulate a large amount of thioglucoside glucohydrolases (TGGs, also known as myrosinases) in their vacuoles. Myrosinases convert their substrates, glucosinolates, into toxic compounds when herbivories and pests attack plants. In this review, we highlight the similarities and differences between myrosin cells and vascular cells/guard cells (GCs) because myrosin cells are distributed along vascular cells, especially the phloem parenchyma, and myrosin cells share the master transcription factor FAMA with GCs for their cell differentiation. In addition, we analyzed the overlap of cell type-specific genes between myrosin cells and GCs by using published single-cell transcriptomics (scRNA-seq) data, suggesting significant similarities in the gene expression patterns of these two specialized cells.

## Physiological Roles of the Myrosinase–Glucosinolate System

Idioblasts are cells with abnormal shapes, sizes, and contents (proteins and metabolites) (Foster, 1956). Myrosin cells are Brassicales-specific idioblasts. In model plants, *Arabidopsis thaliana*, myrosin cells are distributed along veins in the aerial parts of the plants without hypocotyls. Myrosin cells are named after storage proteins, myrosinases, which are called “myrosin grain” and thioglucoside glucohydrolase (TGG) (Rask et al., 2000; Andreasson et al., 2001). The substrates of myrosinases are glucosinolates, which are sulfur-rich compounds in plants and accumulate at other types of specialized cells called S-cells (Koroleva et al., 2000; Shroff et al., 2008; Burow and Halkier, 2017; Nintemann et al., 2018). Under normal developmental conditions, enzymes and substrates never meet each other because they are separated into different cell types. After the cell collapses by the attack of herbivories, myrosinase cleaves the bond between sulfur and glucose in glucosinolates to produce the toxic compounds, isothiocyanates (Shirakawa and Hara-Nishimura, 2018). This defense strategy was named “mustard oil bomb” and is one of the critical factors for the habitat range of Brassicales plants (Prasad et al., 2012). In a model plant, *A. thaliana*, two kinds of myrosinases, TGG1 and TGG2, are stored in myrosin cells. Double knockout mutants of *TGG1* and *TGG2* have exhibited weak resistance against herbivories compared with the wild-type (Barth and Jander, 2006). Unlike *TGG2*, it is well known that *TGG1* is also expressed in guard cells (GCs) (Zhao et al., 2008). However, the role of the myrosinase–glucosinolate system in GCs was unknown until recently. Salehin et al. (2019) showed that the myrosinase–glucosinolate system is required for the closure of stomata under drought conditions. These results suggested that the myrosinase–glucosinolate system has different functions in two different specialized cells.

Thioglucoside glucohydrolases are defined by the conserved glutamine that is required for binding to glucosinolates. The conserved residue is replaced by glutamic acid in the atypical myrosinases, PENETRATION2 (PEN2) and PYK10 (Matsushima et al., 2003; Bednarek et al., 2009). They prefer indole glucosinolates to aliphatic glucosinolates. For more detailed information about the function and evolution of atypical myrosinases, see Nakano et al. (2014); Pastorczyk and Bednarek (2016), and Nakano et al. (2017).

## Anti-Myrosinase–Glucosinolate Strategies and Reuse of Glucosinolates

During evolution, herbivories evolved the resistance and secondary use of the myrosinase–glucosinolates system. Diamondback moth is a crucifer specialist insect and produces glucosinolate sulfatase (GSS) to detoxify glucosinolates (Ratzka et al., 2002). GSS hydrolases glucosinolates to produce desulfo-glucosinolates, which myrosinases cannot cleave. The evolution of GSS during the battle between insects and plants is an open question.

Other insects accumulate plant toxins, glucosinolates, for defense against predators. The specialist herbivorous insect *Phyllotreta striolata* (flea beetle) ingests glucosinolates and has myrosinases that may cleave glucosinolates from plants (Beran et al., 2014). Recently, it was reported that the horseradish flea beetle *Phyllotreta armoraciae* uses a sugar transporter as a glucosinolate transporter to transfer glucosinolates from the excretory system to the hemolymph (Yang et al., 2021). It is an interesting question how and why flea beetles start to use glucosinolates for their defense strategies.

## Myrosin Cells Versus Vascular Cells

Myrosin cells are distributed along veins, especially the phloem (Shirakawa and Hara-Nishimura, 2018; Shimada et al., 2018). Myrosin cells contact directly with phloem parenchyma. During development, myrosin cells never encounter veins, resulting in two networks, the network of veins and myrosin cells, which are wired coordinately (parallel organization and alignment). This observation provoked the question of whether myrosin cells differentiate from vascular precursor cells (procambium cells). Two groups compared the spatiotemporal expression patterns of a myrosin cell reporter and a procambium reporter and showed that myrosin cells do not differentiate from procambium cells and, rather, directly differentiate from ground meristem cells, which are stem cell-like cells in inner tissue (Li and Sack, 2014; Shirakawa et al., 2016b). Ground meristem cells are also mother cells of mesophyll cells and procambium cells. How plants coordinate the development of myrosin cells and vascular cells remains an open question. Polar auxin transport (PAT) is required for the proper development of both vascular cells and myrosin cells, suggesting that auxin may coordinate the development of both cell types. Interestingly, mutants of *SYNTAXIN OF PLANTS 22* (SYP22) exhibited a less vascular network than wild-type and, in contrast, had more myrosin cells than the wild-type (Ueda et al., 2006; Shirakawa et al., 2009, 2010, 2014a). SYP22 is required for PAT in leaf primordia through the endocytosis of the auxin efflux carrier PIN-FORMED 1 (PIN1) (Shirakawa et al., 2014c). *syp22* failed to canalize auxin resulting the abnormal distribution of auxin. Taken together, we hypothesized that high levels of auxin induce the vascular differentiation and low levels of auxin induce the differentiation of myrosin cells. Different dosages of auxin may regulate the development of two specialized cells.

Why do myrosin cells distribute along leaf veins? First, S-cells are distributed along the primary veins. Plants need to develop myrosin cells close to S-cells to efficiently produce toxic compounds when herbivories eat them. This may work as a costless defense system, protecting the lifeline of plants without the loss of photosynthetic organs. Second, myrosin cells may need to communicate with vascular cells, especially phloem cells, to exchange nutrients and metabolites. Consistent with this hypothesis, SUGAR TRANSPORTER PROTEIN 8 is specifically expressed in myrosin cells (Rottmann et al., 2018). Like the wiring of the vein network and myrosin cell network, two networks, the nervous system and blood vessel network, are wired in animals and are called “neurovascular links” (Walchli et al., 2015). The wiring of networks of vascular cells and myrosin cells (named myrovascular links) may be a good model for research on the coordination of the two networks. Future studies may identify new roles of myrosin cells in the context of communication with vascular cells, which may be independent of S-cells.

## Myrosin Cells Versus Guard Cells

The basic helix–loop–helix transcription factor FAMA was identified as a master transcription factor for the differentiation of myrosin cells from ground meristem cells (Li and Sack, 2014; Shirakawa et al., 2014b, 2016a). FAMA is also known as the master TF for the transition from guard mother cells (GMCs) into GCs (Ohashi-Ito and Bergmann, 2006). Sister TFs, SPEECHLESS (SPCH) and MUTE, regulate the transition from protodermal cells into meristemoids and the transition from meristemoids into GMCs, respectively (MacAlister et al., 2007; Pillitteri et al., 2007; Lau and Bergmann, 2012; Han and Torii, 2016). Recently, it was shown that MUTE directly activates *FAMA* in stomatal lineage cells (Han et al., 2018). However, in inner tissues, MUTE is not required for the expression of *FAMA*, suggesting that other TF(s) activate the expression of *FAMA* in inner tissues (Shirakawa et al., 2014b). The distribution of *FAMA*-expressing cells in inner tissues was changed by treatment with PAT inhibitors and in mutants of PAT, including *syp22* (Li and Sack, 2014; Shirakawa et al., 2014c). Auxin response factors (ARFs) may activate *FAMA* in inner tissues.

**FIGURE 1:**
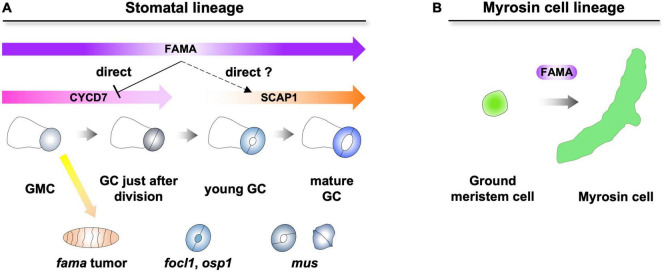
Dual role of FAMA in epidermis and inner tissues. **(A)** FAMA regulates the final division of guard mother cells (GMCs) and the differentiation of guard cells (GCs). FAMA directly represses the expression of *CYCD7* and potentially regulates the expression of *SCAP1*. Loss of *FAMA* triggers the ectopic divisions of GMCs, resulting in the formation of *fama* tumors. In *focl1* and *osp1*, the pores of stomata are covered by membranous cuticular material. *mus* exhibits skewed GC (left) and unopen GC (right). **(B)** FAMA regulates the differentiation of ground meristem cells into myrosin cells in inner tissues.

The downstream FAMA has been well studied in the stomatal lineage (Hachez et al., 2011; Weimer et al., 2018). In the stomatal lineage, FAMA inhibits the ectopic divisions of GMCs and promotes the differentiation of GCs (Ohashi-Ito and Bergmann, 2006). One of the D-type cyclins, CYCD7, is directly repressed by FAMA to inhibit ectopic divisions of GMCs (Figure 1A; Weimer et al., 2018). Although other downstream factors were identified by transcriptome analysis (Hachez et al., 2011), it was still unclear which direct targets of FAMA differentiate from GMCs to GCs. One of the candidates is DNA-binding with one finger (DOF) TF, STOMATAL CARPENTER 1. *SCAP1* is upregulated in inducible FAMA-overexpression lines (Hachez et al., 2011). *SCAP1* is expressed from young GCs to mature GCs, suggesting that the expression window of *SCAP1* fits the later expression window of *FAMA* (Figure 1A; Negi et al., 2013; Lopez-Anido et al., 2021). In addition, half of GCs in *scap1* mutants exhibited skewed morphologies (Negi et al., 2013). *SCAP1* is a potential direct target of FAMA in young GCs. However, the stomatal phenotypes of *scap1* were much weaker than those of *fama*. Other direct target(s) of FAMA must exist and cooperatively promote the differentiation of GCs with SCAP1.

In addition to TFs, a leucine-rich repeat receptor-like kinase (MUSTACHES/MUS), a structural protein [FUSED OUTER CUTICULAR LEDGE1 (FOCL1)] and enzymes [POLYGALACTURONASE INVOLVED IN EXPANSION3 (PGX3) and OCCLUSION OF STOMATAL PORE 1 (OSP1)] that are involved in the formation of pores were identified over a decade (Keerthisinghe et al., 2015; Hunt et al., 2017; Rui et al., 2017; Tang et al., 2020). *mus* exhibited skewed GC and unopen GC, suggesting that MUS receives unknown ligands that coordinate the bilateral symmetry of GC. *focl1* and *osp1* showed similar phenotypes in that the pores of stomata were covered by membranous cuticular materials. FOCL1 is a secreted cell wall structural protein, and OSP1 is a GDSL lipase. It is an interesting question how FOCL1 and OSP1 interact genetically and biochemically. PGX3 is required for the formation of the proper pore size. It is still an open question whether the expression of these factors is regulated by FAMA.

Compared with stomata, key factor(s) of the differentiation of myrosin cells, which are downstream of FAMA, have not yet been identified (Figure 1B). Only one of the myrosinases, TGG1, has recently been reported to be a direct target of FAMA (Feng et al., 2021). Overall, key factor(s) that promote the differentiation of two specialized cells after FAMA remain enigmatic. In addition to downstream factors of FAMA, several interaction partners of FAMA have been identified (Mair et al., 2019). One of them, SCREAMs, is required for the differentiation of stomata and myrosin cells (Kanaoka et al., 2008; Shirakawa et al., 2014b). Other factors may have specific developmental/physiological functions in one of two specialized cells.

## Commonality Between Myrosin Cells and Guard Cells

The FAMA-SCRM complex is a common master regulator of the differentiation of both myrosin cells and GCs. Therefore, it is expected that the two cell types may share gene expression patterns. Very recently, transcriptome analysis of *Arabidopsis* leaves was performed at single-cell resolution (scRNA-seq) (Berrío et al., 2021). Surprisingly, during the analysis, the unsupervised clustering of scRNA-seq data recognized myrosin cells and GCs as a single cluster. In addition, then, combined with the known markers (an epidermal marker was only expressed at GCs, not at myrosin cells), the authors divided them into two different clusters. Consistent with the previous experiment with reporter lines, *TGG1* was expressed in both cell types, and *TGG2* was exclusively expressed in myrosin cells in scRNA-seq, suggesting that scRNA-seq with manual clustering successfully separated the two cell types.

**FIGURE 2:**
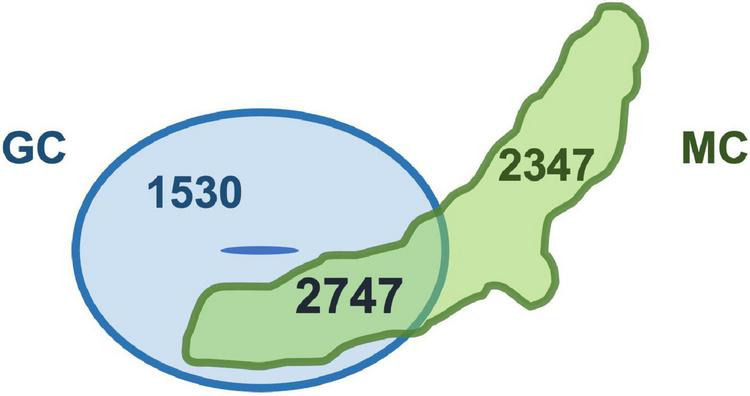
Two specialized cells share thousands of genes. Venn diagram of genes specifically expressed in guard cells (GC) and myrosin cells (MC). Original data were reported in Berrío et al. (2021).

These results suggest that the gene expression patterns of the two types of specialized cells may be quite similar. Indeed, we compared cell type-specific genes of two specialized cells, and the analysis revealed that the commonly expressed genes were more than 50% of genes expressed in each cell type (myrosin cells 54%; GCs 64%) (Figure 2). This result indicates that unknown common function(s) may exist in two specialized cells. In addition, some myrosin cells expressed vascular markers, suggesting that myrosin cells can be segmentized into more small groups and that some of them may have features of vascular cells (Berrío et al., 2021). Taken together, scRNA-seq is a powerful tool for research on cell fate determination and differentiation.

## Perspectives

In this review, we discuss the functional and developmental link between myrosin cells and vascular cells and GCs. The link between myrosin cells and vascular cells is hypothesized from anatomical research, and the unexpected link between myrosin cells and GCs is hypothesized from the discovery of master transcription factors. These hypotheses were partly supported by recent scRNA-seq data (Figure 2). These hypotheses may be connected with findings of the new physiological role of myrosin cells.

The downstream factors of FAMA must provide important information to answer this question. During this decade, some direct targets of FAMA were identified. However, the whole network of FAMA downstream is still largely unknown. By using inducible overexpression lines, RNA-seq analysis with sampling at multiple time points may be useful because FAMA is continuously expressed during the development of GCs (Lopez-Anido et al., 2021), and by using such methods, downstream targets of SPCH and MUTE have been identified (Lau et al., 2014; Han et al., 2018). In addition to classical transcriptome analysis, scRNA-seq is a useful tool not only for the identification of cell type-specific genes but also for the reconstruction of cell lineages (Lopez-Anido et al., 2021). Combined with stage-specific fluorescent reporters, fluorescence-activated cell sorting (FACS), and scRNA-seq, it may be possible to reveal the gradual change in gene expression patterns of myrosin cells from the beginning to the maturation of cell differentiation (lineage tracing).

Moreover, in the plant research field, small molecules with various biological functions have been recently identified (Nemhauser and Torii, 2016; Ziadi et al., 2017; Shirakawa et al., 2021). For example, the chemical compound bubblin increased the number of GCs (Sakai et al., 2017). By transient treatment and dosage control, small molecules can overcome the genetic redundancy and lethality of gene functions. It could be possible to identify the molecules that manipulate the number and distribution of myrosin cells and convert the identity of myrosin cells into GCs/vascular cells and vice versa.

The physiological function and developmental program of idioblast myrosin cells are largely unknown. Future works will shed light on the comprehensive molecular network of the function and development of myrosin cells. This information may be connected with the research field of applied plant science.

## Author Contributions

MS and MT analyzed the scRNA-seq data and described the figures. MS and TI wrote the manuscript. All authors read and approved the final version of the manuscript.

## Conflict of Interest

The authors declare that the research was conducted in the absence of any commercial or financial relationships that could be construed as a potential conflict of interest.

## Publisher’s Note

All claims expressed in this article are solely those of the authors and do not necessarily represent those of their affiliated organizations, or those of the publisher, the editors and the reviewers. Any product that may be evaluated in this article, or claim that may be made by its manufacturer, is not guaranteed or endorsed by the publisher.
